# Risk of Poisoning from Garden Plants: Misidentification between Laurel and Cherry Laurel

**DOI:** 10.3390/toxins14110726

**Published:** 2022-10-24

**Authors:** Paola Malaspina, Federica Betuzzi, Mariarosaria Ingegneri, Antonella Smeriglio, Laura Cornara, Domenico Trombetta

**Affiliations:** 1 Department of Earth, Environment and Life Sciences (DISTAV), University of Genoa, Corso Europa 26, 16132 Genoa, Italy; 2 Department of Chemical, Biological, Pharmaceutical and Enviromental Sciences (ChiBioFarAm), University of Messina, Viale Ferdinando Stagno d’Alcontres 31, 98166 Messina, Italy

**Keywords:** *Laurus nobilis*, *Prunus laurocerasus*, toxic plants, edible plants, microscopy, phytochemistry

## Abstract

The misidentification between edible and poisonous plants is an increasing problem because of the new trend to collect wild plants, especially by amateur collectors who do not have the botanical skills to distinguish between edible and toxic species. Moreover, morphologically similar species are sometimes responsible for accidental contamination or used in the intentional adulteration of products for human and animal consumption. *Laurus nobilis* L. (laurel) and *Prunus laurocerasus* L. (cherry laurel) are typical ornamental shrubs of the Mediterranean region. Laurel is considered a non-toxic plant, widely used as flavorings. Conversely, cherry laurel leaves, morphologically similar to those of laurel, contain toxic cyanogenic glycosides. Considering this, the aim of this study was to carry out an in-depth evaluation of laurel and cherry laurel leaves by using light and scanning electron microscopy coupled with three step phytochemical analyses (qualitative and quantitative colorimetric assays and liquid chromatography). This allowed to highlight the distinguishing features of plant species investigated features such as the venation pattern, presence/absence of nectaries, calcium oxalate crystals, secretory idioblasts, and cyanogenic glycosides. Concluding, this multidisciplinary approach can be useful for the identification of plants but also fragments or pruning residues containing cyanogenic glycosides, in quality control tests, intoxications, and criminal cases.

## 1. Introduction

Plant poisoning of humans and animals is a growing problem worldwide [1] because lots of species, in particular belonging to different families, including Solanaceae, Papaveraceae, Apocynaceae, Ranunculaceae, Liliaceae, and others, produce harmful secondary metabolites, which can be found in the whole plant or in specific organs, such as leaves or fruits. The toxic potential of plants depends on the species involved, the amount ingested or rubbed on the skin, the parts consumed, and sometimes the plant growth stage [2]. 

Based on the annual reports drawn up by the Poison Control Center of Niguarda Hospital in Milan, a serious problem concerning human intoxications is the misidentification between edible and toxic plants [3]. An increasing number of people, lacking in proper botanical knowledge, is indeed getting into the habit of picking up outdoor plants to prepare foods or to treat ailments, unaware of the possible dangers related to the presence of toxic plants [4]. Moreover, a great part of the herbal products sold all over the world includes contaminants, substitutes, or filler species, which can cause serious damage to health [5]. A well-known example is the contamination or adulteration of Chinese star anise (*Illicium verum* Hook. f.), rich in healthy properties, with the related species, morphologically similar, Japanese star anise (*Illicium anisatum* L.), which instead shows neurological and gastrointestinal toxicity [6]. Another problem, pointed out by Moro et al. [7] is that children’s and pets’ health is often threatened by the presence of hazardous ornamental species in school gardens, public parks, and houses, due to a missing risk evaluation. Kids and domestic animals can indeed ingest parts of plants for curiosity or gaming. Regarding farm animals, poisoning is generally a result of a feed contamination or a lack of other food supplies [2]. In addition, the presence of biomass from pruning removal of ornamental plants containing toxic compounds is a risk factor for livestock [8]. 

Several cases of accidental contamination or intentional adulteration from foreign plant material or products with ambiguous nomenclature, threatening public health, have been frequently reported [5,9,10]. Concerning the Mediterranean region, accidental misidentification due to inexperience occurs with several species, and an important case of wrong identification involves *Prunus laurocerasus* L., which can be frequently collected instead of *Laurus nobilis* L. [3,11]. The first one is an evergreen shrub containing a high quantity of cyanogenic glycosides such as prunasin, sambunigrin, and amygdalin. In particular, the leaves contain 1% to 2.5% prulaurasin (racemic mixture of both prunasin and sambunigrin) as the major components [12]. On the contrary, *L. nobilis* is an evergreen Mediterranean shrub or small tree, whose leaves are very rich in polyphenols such as flavonoids, phenolic acids, proanthocyanidins, and lignans, and which are traditionally used as a cooking flavour and in folk medicine due to their beneficial health effects [13]. 

Therefore, the purpose of this work was to carry out a multidisciplinary study on *P. laurocerasus* and *L. nobilis*, by using a macro- and micro-morphological characterization of the leaves coupled with three-step phytochemical analyses (qualitative and quantitative colorimetric assays and liquid chromatography analysis), to highlight the distinguishing features that can allow to correctly identify both plants. The data collected can ensure a more detailed quality control of plant material, even if it is crushed or fragmented. This is a critical step of the food supply chain because the correct identification of the species intended for both human and animal consumption is important in the healthcare sector. This kind of approach is also useful for recognizing plant remnants found in both human and animal biological samples, to figure out which species had caused the intoxication and so proceed accordingly in the shortest possible time.

## 2. Results

### 2.1. Macro- and Micro-Morphological Analyses

**Figure 1:**
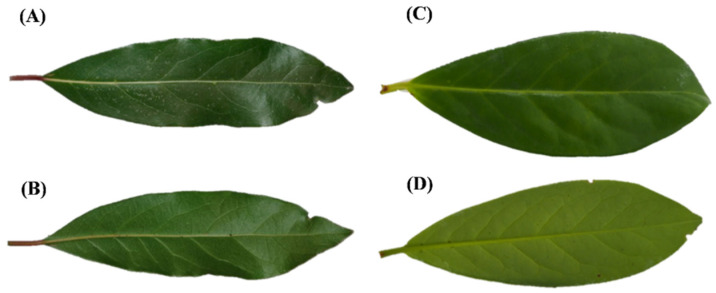
Macro-morphology of the leaf: (**A**,**B**)—*L. nobilis*; (**C**,**D**)—*P. laurocerasus*; (**A**,**C**)—upper surface; (**B**,**D**)—lower surface.

*Laurus nobilis* and *P. laurocerasus* leaves are both leathery, petiolate, dark green, shiny on the adaxial surface, and pale green and dull on the abaxial one. Concerning shape, the first are oblong and acuminate (Figure 1A,B), while the second ones are obovate-lanceolate with a maximum width near the apex (Figure 1C,D). Other distinctive differences concern the petiole, which is red in laurel and green in cherry laurel, and the edge, which is slightly undulated in laurel and serrated in cherry laurel (Figure 1). 

**Figure 2:**
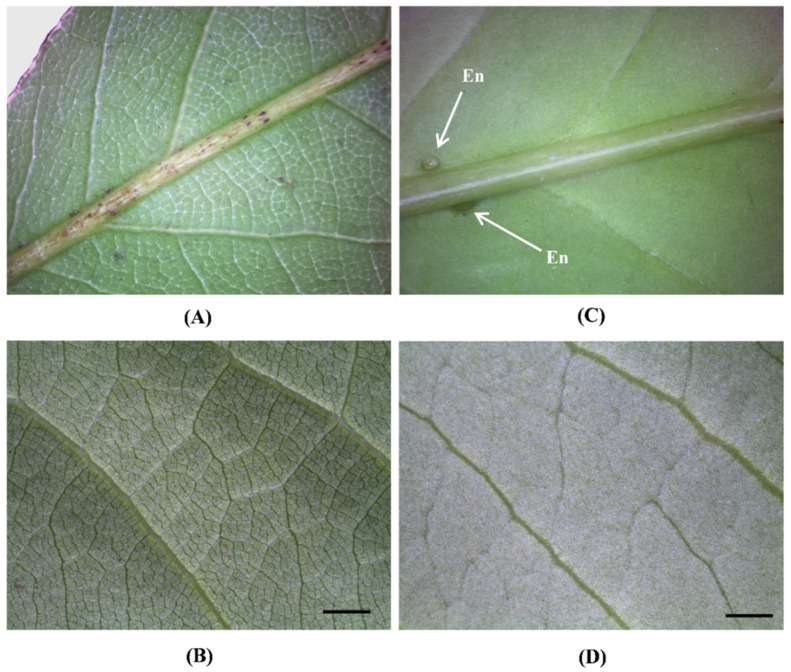
Macro-morphological characteristics of the leaf lower surface: (**A**,**B**)—*L. nobilis*; (**C**,**D**)—*P. laurocerasus*. LM: leaf base near the petiole (**A**), leaf base near the petiole with extrafloral nectaries on both sides of the midrib (**C**), detail of the different venation pattern (**B**,**D**). En: Extrafloral nectaries (arrows). Bar = 2 mm.

However, the main macro-morphological distinguishing feature is the absence of nectary glands in *L. nobilis* leaf (Figure 2A), while on the contrary extrafloral nectaries are well visible on the lower surface of *P. laurocerasus*, located near the petiole and on both sides of the midrib (Figure 2C).

Moreover, if rubbed, *P. laurocerasus* leaves release an almond scent due to the presence of cyanogenic glycosides, while *L. nobilis* leaves have a characteristic camphoraceous fragrance due to the essential oil. A different venation network is already visible at the macroscopic level (Figure 2A,C), which is highlighted in greater detail at the micro-morphological level.

**Figure 3:**
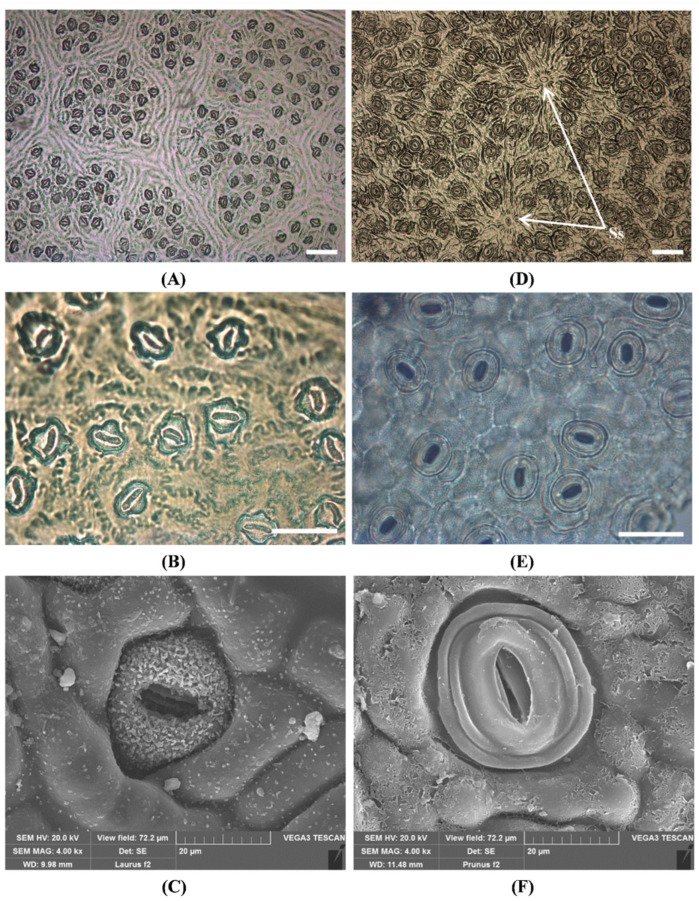
Stomata in the lower epidermis of the leaf: (**A**–**C**)—*L. nobilis*; (**D**–**F**)—*P. laurocerasus*. LM: paracytic stomata enclosed in areoles (**A**,**B**), solitary stomata (**D**), anomocytic stomata (**E**). SEM: focus on a sunken paracytic stomata (**C**), focus on an anomocytic stomata (**F**). Ss: solitary stomata (arrows). Bar = 100 micron (**A**,**D**). Bar = 500 micron (**B**,**E**).

*Laurus nobilis* shows a reconnecting type of reticulation pattern, with tertiary and quaternary veins that enclose areoles (Figure 2B and Figure 3A). On the contrary, quaternary veins are instead absent in *P. laurocerasus* (Figure 2D and Figure 3D).

In both species, stomata are confined to the abaxial surface. Concerning *L. nobilis*, the stomatal apparatus is typical of the Lauraceae family, with rubiaceous stomata, mostly sunken below the surface, and subsidiary cells not easily recognizable (Figure 3B,C). The abaxial epidermis consists of elongated cells with sinuous-beaded anticlinal walls (Figure 3B). Differently, *P. laurocerasus* shows anomocytic stomata with clearly visible kidney-shaped guard cells and subsidiary cells, similar to the epidermal ones (Figure 3E). The outer stomatal ledges are surrounded by one or several rings formed by cuticular folds (Figure 3F), which can sometimes overlap other epidermal cells. There are also few anisocytic stomata, with three subsidiary cells of different sizes, and solitary stomata clearly distinct from normal ones but similar in size (Figure 3D, arrows). The abaxial epidermis presents polygonal cells with thick walls. 

**Figure 4:**
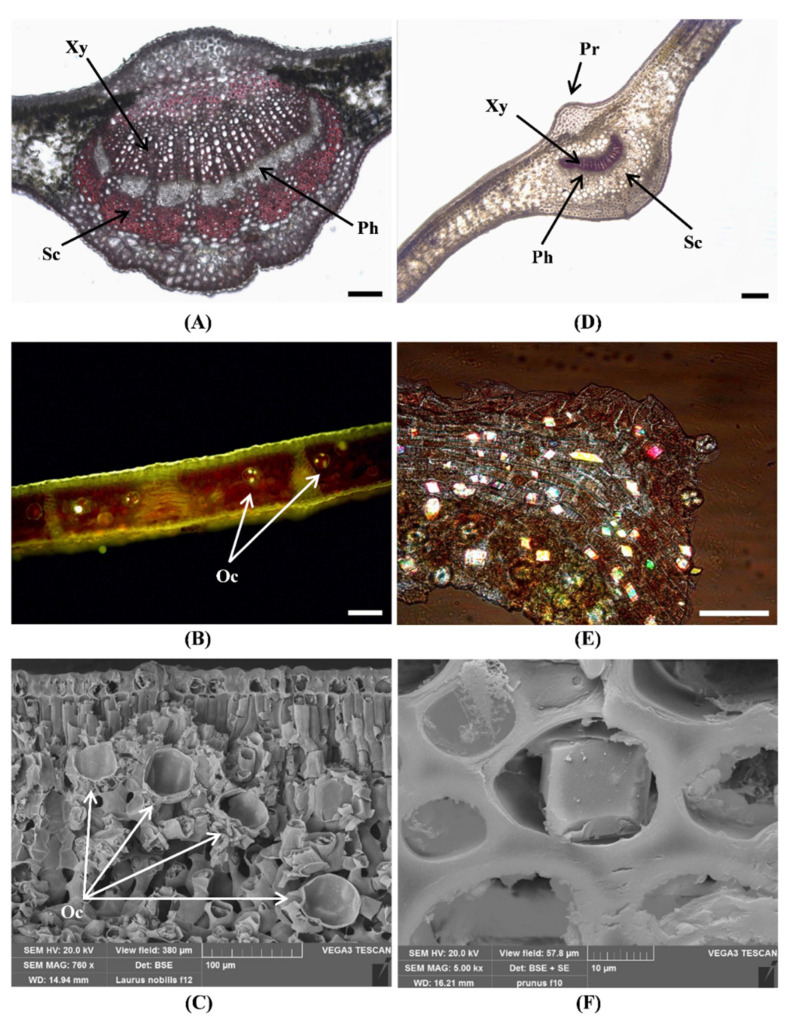
Micro-morphological characteristics of the leaf of *L. nobilis* (**A**–**C**) and *P. laurocerasus* (**D**–**F**). (**A**,**D**)—Phloroglucinol-HCl; (**B**)—Fluorol Yellow 088; (**E**)—Polarized light; (**C**,**F**)—SEM. *L. nobilis*: leaf cross section with slight collenchymatous protrusion (**A**); secretory idioblasts containing droplets of essential oil stained by Fluorol Yellow (**B**); cross-section showing secretory idioblasts without oil content, scattered in the mesophyll (**C**). *P. laurocerasus*: leaf cross section with evident collenchymatous protrusion (**D**); epidermal peel showing many calcium oxalate crystals along the veins (**E**); a single calcium oxalate crystal, at high magnification, within a collenchyma cell (**F**). Xy: xylem, Ph: phloem, Sc: sclerenchyma, Pr: collenchymatous protrusion, Oc: oil cell (arrows). Bar = 100 micron (**A**,**B**,**E**). Bar = 200 micron (**D**).

The midrib is adaxially convex in both species (Figure 4A,D).

In cross section, a collenchyma protrusion situated above the leaf midrib is clearly visible in *P. laurocerasus* (Figure 4D), while a less evident protrusion is present in the leaf. of *L. nobilis* (Figure 4A).

Furthermore, several secretory idioblasts containing droplets of essential oil (Figure 4B) are distributed in the mesophyll of *L. nobilis* and are responsible for its typical aroma. These oil cells are spherical and large, with a mean diameter of 44.3 μm ± 4 (n = 12) (Figure 4B,C).

**Figure 5:**
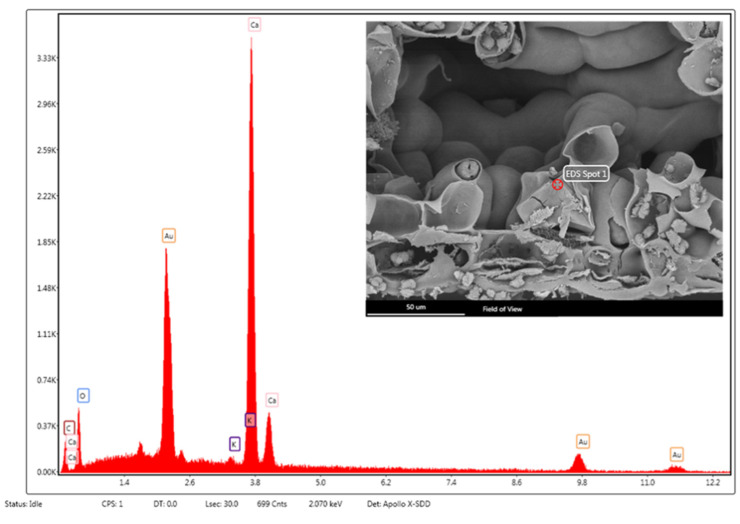
SEM–EDS structural characterization of a prismatic crystal of calcium oxalate in *P. laurocerasus* leaf. The peaks denoted by “Au” correspond to the gold sputter coating of the sample.

In *P. laurocerasus* leaves, many calcium oxalate prismatic crystals are visible, mainly distributed along the veins (Figure 4E), in the palisade parenchyma near the upper epidermis, and in the cells of the collenchyma (Figure 4F) surrounding the midvein. Crystal composition is confirmed by SEM-EDS analysis (Figure 5).

### 2.2. Phytochemical Analysis

In this study, addressing both poisoning due to collection of spontaneous plants and forensic cases, the sample preparation was developed from fresh and dry plant material. The extraction method adopted (70% ethanol by sonication for 30 min at RT) was chosen among those tested because it allowed the extraction of the highest concentration of prunasin from both fresh and dry *P. laurocerasus* leaves, showing accurate and reproducible results, without any statistically significant difference between fresh and dry leaves of *P. laurocerasus* (Table S1).

**Figure 6:**
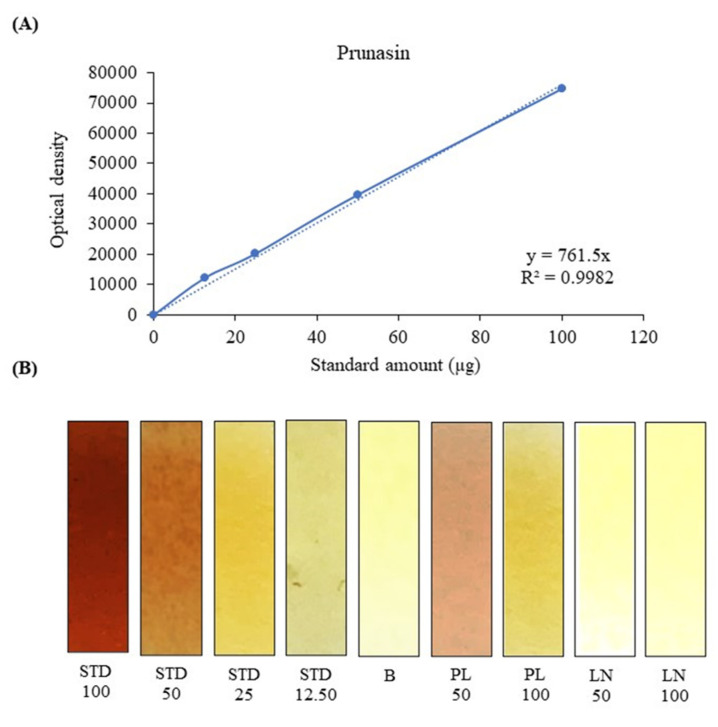
Semi-quantitative determination of cyanogenic glycosides by the picric acid method: (**A**) prunasin calibration curve, built by plotting the densitometric readings of the test strips against the standard amount (µg) tested. (**B**) Representative figure of the test strips, after treatment with different volumes (100, 50, 25, 12.50 µL) of the reference standard prunasin (STD, 1 mg/mL), blank (**B**) and with two different volumes (50 and 100 µL) of *P. laurocerasus* and *L. nobilis* leaf extracts (PL and LN, 1 mg/mL).

After this, two rapid screening methods were developed, the first qualitative and semi-quantitative, the second one spectrophotometric. The first, that is the picric acid method, was developed for the determination of cyanogenic glycosides by using prunasin as a reference standard, with the intention of developing a sort of kit, which would allow to discriminate with a simple colorimetric reaction (positive/negative) between the presence or absence of these toxic metabolites. As can be seen from Figure 6B, by comparing the test strips of the sample (PL, Figure 6B) with those treated with the reference standard prunasin (STD, Figure 6B), it is easy to recognize the presence of cyanogenic glycosides in comparison with the strip treated with 70% ethanol only (B, Figure 6B). Furthermore, the strip treated with the laurel extract does not give any color-change, showing a behavior superimposable to the blank (LN, Figure 6B).

**Figure 7:**
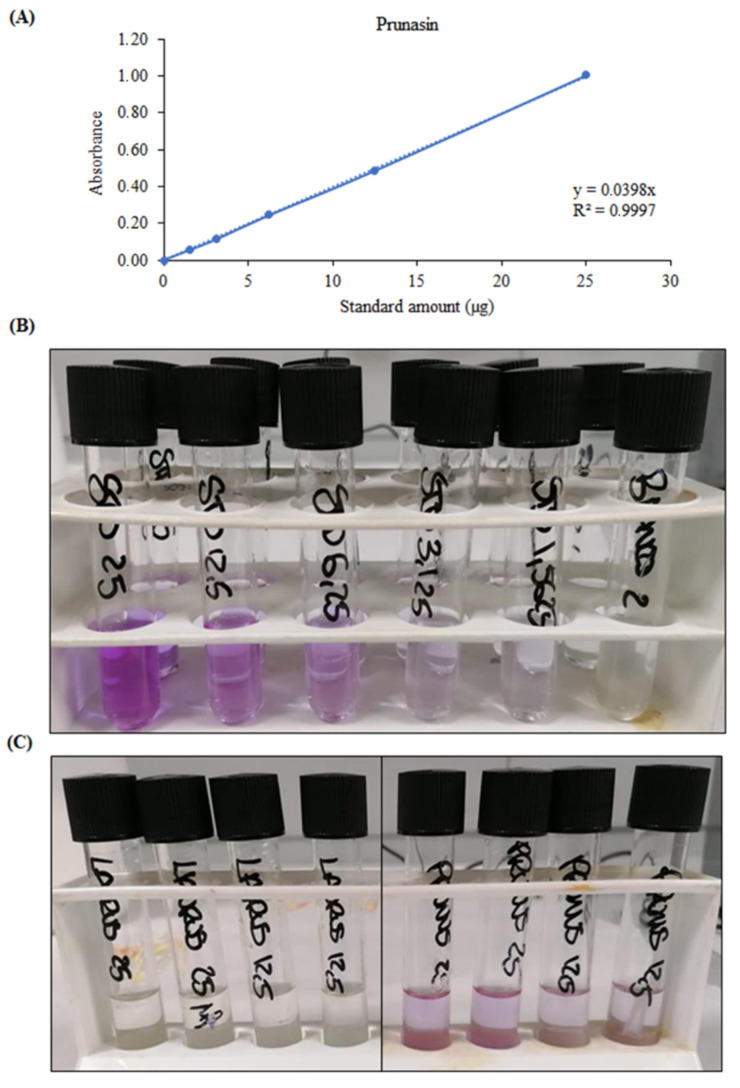
Spectrophotometric determination of cyanogenic glycosides with the pyridine–barbituric acid method: (**A**) calibration curve of prunasin built by plotting the absorbance against the standard amount (µg) tested; (**B**) representative figure of the colorimetric test after treatment with different volumes (25, 12.50, 6.25, 3.125, 1.5625, 0 µL) of the reference standard prunasin (1 mg/mL); (**C**) representative figure of the colorimetric test after treatment with different volumes (25 and 12.50 µL) of *L. nobilis* and *P. laurocerasus* leaf extracts (1 mg/mL).

This method had already been adopted previously for the determination of cyanogenic glycosides in American elderberry using amygdalin as a reference standard [14]. In this study, a semi-quantitative analysis acquiring the photos of the colorimetric reaction and processing them without any modification with the ImageJ software, was also carried out. By plotting the optical density of the test strips against the quantity of reference standard used, it was possible to build a standard calibration curve that gave good results in terms of linearity (R^2^ = 0.9982) in the concentration range considered (STD 0–100 µg, Figure 6A), according to the Beer–Lambert law. This made it possible to extrapolate the concentration of cyanogenic glycosides in the samples under examination as 3.88 ± 0.272 g PE/100 g fresh weight (FW), with results very similar to those obtained with the following spectrophotometric test (3.33 ± 0.141 g PE/100 g FW). Indeed, also in this case, using the pyridine-barbituric acid method, previously developed by Kobaisy et al. [15] for the determination of cyanogenic glycosides in flaxseed, and here modified for the determination of the same compounds in the plant matrices under examination, it was possible to develop a sensitive, accurate and reproducible reaction. As can be seen from Figure 7B, the standard prunasin as well as the cherry laurel samples take a very intense purple color, making the positivity of the reaction easily recognizable. Furthermore, plotting the absorbances recorded at 585 nm, with respect to the amount of standard added to the test tubes (0–25 µg), a calibration curve with an excellent linearity (R^2^ = 0.9997) was obtained. This allows to adequately calculate the concentration of cyanogenic glycosides in cherry laurel samples expressed as prunasin equivalents (3.33 ± 0.141 g PE/100 g FW). Also in this case, despite the greater sensitivity of the test, no interference was found with the laurel extract, which showed a superimposable behavior to that of blank (Figure 7C).

**Figure 8:**
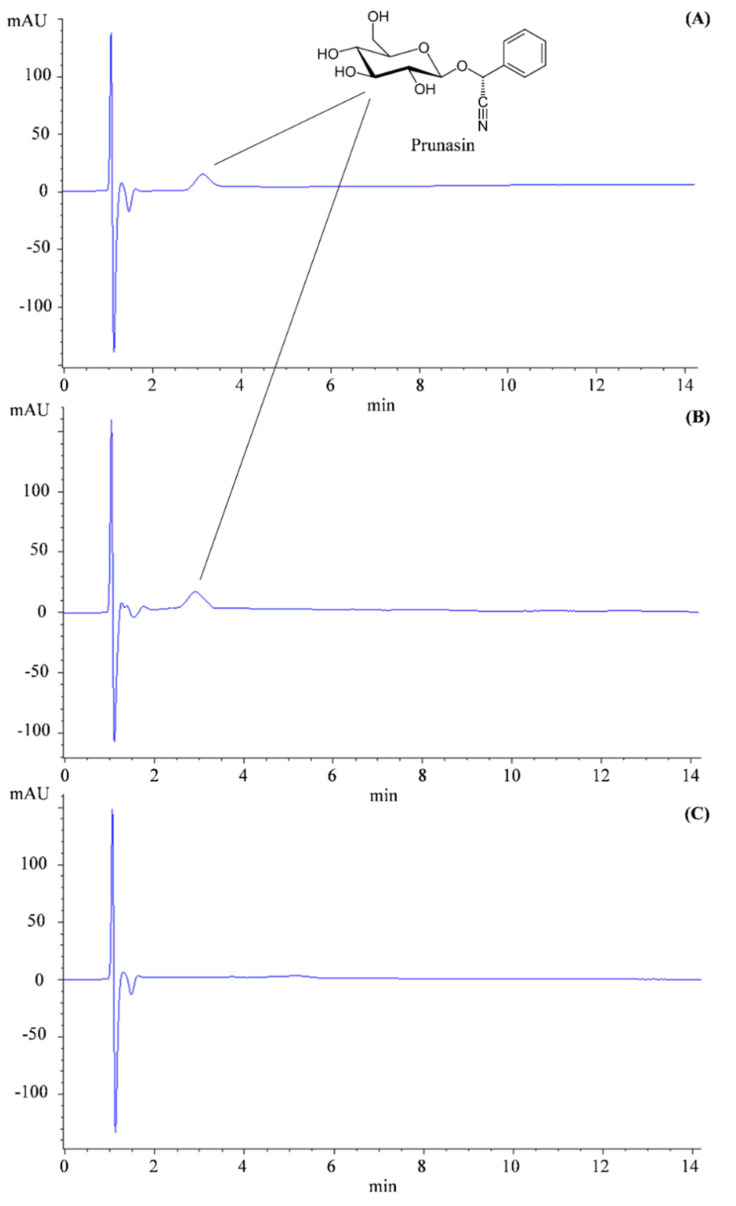
Representative HPLC–DAD chromatogram acquired at 220 nm of the reference standard prunasin 20 µg/mL (**A**), leaf extract (1 mg/mL) of *P. laurocerasus* (**B**) and leaf extract (5 mg/mL) of *L. nobilis* (**C**).

Finally, with reference to forensic cases, in which it is more difficult to detect the presence of these substances in biological samples without an adequate analytical sensitivity, a rapid quali-quantitative analysis has been developed by HPLC-DAD. As can be seen from Figure 8, which shows the representative chromatograms of the standard prunasin (Figure 8A), of the cherry laurel extract (Figure 8B) and of the laurel extract (Figure 8C) acquired at 220 nm, the analytical method resulted in a fast and simple detection of prunasin without analytical interference.

## 3. Discussion

Laurel and cherry laurel are both commonly cultivated in the Mediterranean region as ornamental trees in home gardens, urban green areas, and grazing fields, mainly for creating hedges. Cherry laurel and laurel have morphologically similar leaves, so that some inexperienced herb gatherers could confuse the two plants’ identities. *L. nobilis* is largely used as flavoring agent or folk remedy [16]. However, the consumption of moderate amounts is recommended because some compounds of the essential oil present in the leaves can easily overcome the blood–brain barrier. Therefore, an excessive uptake of these compounds can cause confusion and neurological disorders in adults and more serious problems in children [17]. Several cases of intoxication due to leaves and fruits of laurel have been reported in Italy, mainly involving children: 102 in the years between 1995 and 2007 [18] and 88 in 2013 [19].

On the other hand, *P. laurocerasus* is a toxic species, because it contains from 1 to 2.5% cyanogenic glycosides [20], such as prunasin in the leaves and amygdalin in the fruits [21]. Generally, intoxications are a consequence of an accidental intake. Both humans and animals are susceptible to any damage resulting from the release of hydrogen cyanide after the ingestion of this plant [20]. However, the human body is capable of inactivating hydrogen cyanide (HCN) in cases of low concentrations, so severe illness is rare [21] and affects especially kids [22]. Instead, ruminants are highly vulnerable to cyanide toxicosis because the rumen microbiota speeds up the hydrolysis of glycosides [23], so hydrogen cyanide is rapidly absorbed into the bloodstream and prevents hemoglobin from releasing its oxygen to the tissues [24]. Regarding Italy, many cases of intoxication by cherry laurel have been reported: 147 between 1995 and 2007 [18] and 28 in 2015 [22]. This plant has also been involved in the poisoning of a dog and two goats during the period 2000–2011 [25]. In the rest of Europe, several deadly cases involving ruminants eating cherry laurel have been reported: in Scotland, 31 sheep died after grazing leaves in a public park [26]; in Germany, two goats died after the ingestion of the leaves present in a green waste [8]; and in Ireland, the death of 36 weanlings was due to the intake of leaves from a hedge situated around the pastureland [23].

Although several studies have described the morphological and anatomical features of both species [11,27,28,29,30], an extensive study, based on both micromorphology and phytochemistry, comparing the leaves of *L. nobilis* and *P. laurocerasus* is lacking.

Micro-morphological analysis of the leaves highlights several distinctive characteristics. Among the identifying features of *L. nobilis*, there is a reconnecting type of reticulation pattern with minor veins enclosing areoles, previously referred also by Blonder et al. [31]. In addition, the typical rubiaceous stomatal apparatus and the large oil glands scattered inside the mesophyll represent diagnostic features. These specific characteristics are useful to identify the laurel even when it is sold in the form of herbal preparations, such as fragmented/powdered leaves.

Differently, *P. laurocerasus* shows anomocytic stomata with outer stomatal ledges surrounded by one or several rings of cuticular folds. According to Pautov et al. [32], sometimes these marginal stomatal rings can overlap even the other epidermal cells. Moreover, some solitary stomata are clearly distinguishable from the normal ones, as previously observed by Boldt and Rank [33]. Finally, in cross section, an evident collenchymatic protrusion situated above the leaf midrib is always clearly visible in *P. laurocerasus*, corresponding to the wedge previously found by Moll and Janssonius [27].

Therefore, even if the two species can be confused by inexpert gatherers, the micro-morphological analysis reveals significant diagnostic differences between their leaves, allowing to correctly identify them. Light microscopy is a quick (few hours) and economical method, but if the samples are very fragmented or partially deteriorated, the SEM support might be more suitable. In this case, the preparation takes more time (several rounds) and higher costs. Our methods can be useful to discriminate different matrices that must be analysed, such as leaf fragments, which can be found in pruning residues, in herbal teas, in biological samples of intoxicated people and animals, or at a crime scene. In the latter cases, phytochemical analyses of the samples can ensure further confirmation of the identity of the species by detecting toxicologically relevant compounds.

In addition, this is the first study that compares laurel and cherry laurel from the phytochemical point of view to avoid misidentification between these two plant species. At this purpose, and to develop and validate properly methods to recognize the toxic plant species, a three-step phytochemical analysis was carried out, passing from a simple qualitative and semi-quantitative analysis to spectrophotometric determination and finally to liquid-chromatography, to identify and quantify the toxic phytochemical marker of the cherry laurel leaf, that is the prunasin [12].

In all three cases, the main purpose was to develop or modify methods which allowed to verify the presence and amount of the cyanogenic glycosides in the leaf of these two plant species, rapidly and rigorously, choosing also the most appropriate method to use, according to the specific case object of investigation. A representative example is that of cases that came to the emergency room following intoxication due to incorrect identification of the plants by inexperienced collectors. In these cases, the development of appropriate, qualitative, or quantitative low-cost methods is auspicial. The two colorimetric assays (picric acid and pyridine–barbituric acid test) developed in the present study are very cheap and fast methods that could be used as first-level assays. On the contrary, the HPLC-DAD method, which requires specialized personnel and higher cost, could be used as confirmation analysis or for detection of these bioactive compounds in biological samples.

In addition to micro-morphological and phytochemical analyses, also molecular methods such as DNA SSR fingerprinting could be used to identify these two species [34], but this type of technique is not, at present, easily accessible by all emergency departments and quality control laboratories.

Our multidisciplinary approach, using techniques already routinely performed at many university and hospital laboratories, let to compare, and correctly identify these two species. In the case of plant material in form of entire or coarsely fragmented leaves, combined macro- and micro-morphological analyses may be enough to discriminate between laurel and cherry laurel. For example, this can represent a valuable tool to identify pruining remnants contaminating the forage or botanical evidence from crime scenes, as in the case reported by Caccianiga et al. [35], where leafly branches of *P. laurocerasus* were used to conceal a victim’s body. On the other hand, phytochemical analyses are more suitable tools to detect the presence of toxic compounds in biological samples, such as stomach content obtained from gastric lavage, blood, and urine.

## 4. Conclusions

The leaves of the two analyzed species show distinctive morphological and phytochemical features and so can be correctly identified by our multidisciplinary approach. Macro- and micro-morphological analyses represent a reliable and simple method to discriminate between cherry laurel and laurel when the leaves are entire or coarsely chopped. For macro-morphological point of view, venation pattern and presence/absence of nectaries are of diagnostic significance, while the main micro-morphological distinctive features are the presence/absence of calcium oxalate crystals and absence/presence of secretory idioblasts. Phytochemical analysis is, instead, a more suitable tool to detect the presence of toxic compounds in human or animal biological samples, such as blood and urine.

Therefore, the complex of our data could be very useful for diagnostic purposes, also considering the high number of intoxication cases due to cherry laurel, in human and animals. Our methods can also be helpful to understand the crime dynamics, as well as to detect adulterants or substituents in quality control tests of food and feed samples.

Finally, one of the future perspectives of our study is to develop a sort of rapid, simple, and cheap kit based on a colorimetric reaction to discriminate between the presence or absence of the toxic metabolite prunasin into a biological sample.

## 5. Materials and Methods

### 5.1. Plant Material

**Figure 9:**
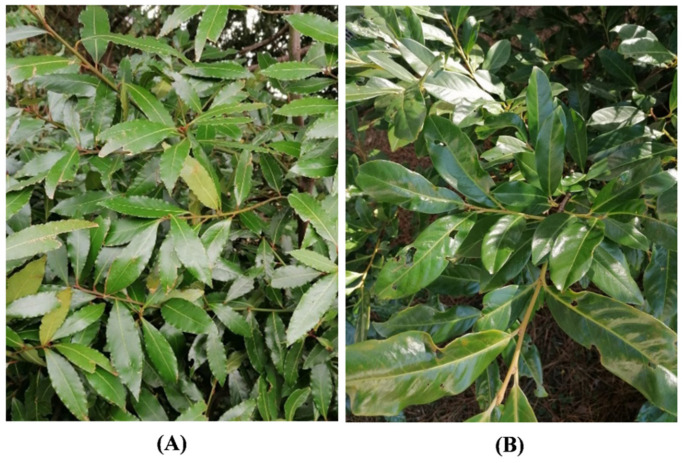
*L. nobilis* (**A**) and *P. laurocerasus* (**B**).

The correct identification of plant species was carried out according to Pignatti et al. [36,37]. *Laurus nobilis* (laurel) is a perennial, aromatic, and dioecious shrub belonging to the Lauraceae family, which grows naturally in the Mediterranean area (Figure 9A). *Prunus laurocerasus* (cherry laurel) is an evergreen outdoor shrub belonging to the Rosaceae family, native to western Asia (Figure 9B). Leaves of both species were collected from Genoa’s Botanical Garden (Liguria, Italy) in March 2021.

### 5.2. Chemicals

Histochemical stains (Phloroglucinol, HCl, Fluorol Yellow 088) and ethanol were purchased from Merck (Darmstadt, Germany), while FineFIX working solution was obtained from Milestone SRL (Sorisole, Bergamo, Italy). Picric acid solution 1.3% H_2_O (saturated), sodium carbonate, LC/MS-ELSD grade prunasin (purity ≥ 90%), β-glucosidase, phosphate buffer, sodium acetate, sodium hydroxide, chloridric acid, chloramine T, barbituric acid, pyridine, and HPLC-grade solvents and reagents (acetonitrile and formic acid) were purchased from Merck (Darmstadt, Germany).

### 5.3. Light Microscopy (LM)

Macro-morphological details of the leaves, such as venation pattern and absence or presence of nectaries, were highlighted by a stereomicroscope (LEICA M205 C—Leica Microsystems, Wetzlar, Germany). 

Micro-morphological characteristics of the leaf anatomy were analyzed by using a transmission light and epifluorescence Leica DM 2000 microscope, coupled with a DFC 320 camera (Leica Microsystems, Wetzlar, Germany). Leaf epidermal peels were obtained from fresh samples with the nail varnish technique or directly tearing off the lower epidermis layer with a tweezer. Cross-sections of fresh leaves were made using a double-edged razor blade and then treated with: Phloroglucinol-HCl to stain lignin and Fluorol Yellow 088 to observe, with UV filter, the light green/yellowish fluorescence indicating the presence of lipid/lipophilic compounds [38]. In addition, polarized light was used to detect the presence/absence of crystals.

### 5.4. Scanning Electron Microscopy (SEM)

Small pieces of the leaves were fixed in FineFIX working solution, left overnight at 4 °C [39], dehydrated for at least 1 h through a graded ethanol series (70, 80, 90, and 100%) and finally dehydrated in CO_2_ using a critical point dryer (K850CPD 2M, Strumenti S.r.l., Roma, Italy). Dried specimens were mounted on stubs using two-sided adhesive carbon tape and sputtered with a 10-nm layer of gold. The samples were examined using a SEM VEGA3-Tescan-type LMU microscope equipped with the X-ray Energy Dispersive System (EDS) (Apollo, Tescan USA Inc., Cranberry Twp, PA, USA), operating at an accelerating voltage of 20 kV. The energy dispersive spectroscopy (EDS) coupled with SEM was used to obtain the elemental composition of crystals. This technique is based on the interaction between the sample and an electron beam, producing characteristic X-rays which are analyzed by a specific detector.

### 5.5. Phytochemical Analyses

#### 5.5.1. Sample Preparation

One-hundred milligrams of both fresh and dry laurel and cherry laurel leaves were powdered by a blade mill (IKA^®^ A11 basic analytical mill, IKA^®^-Werke GmbH & Co. KG, Staufen, Germany). The extraction method was developed and optimized with the aim to maximize the yield in terms of cyanogenic glycosides using food-grade solvents. To this end, several ethanol/water (EtOH/H_2_O) blends were tested (90:10, *v*/*v*; 80:20, *v*/*v*; 70:30, *v*/*v*; 60:40, *v*/*v;* and 50:50, *v*/*v*) at different extraction times (15, 30, 60 min) by sonication in a water bath at different temperatures: room temperature (RT), 30 and 60 °C. The maximum yield in terms of cyanogenic glycosides, expressed as g of prunasin/100 g of fresh and dry leaf extract (FLE and DLE, respectively), was obtained with EtOH/H_2_O 70:30 (*v*/*v*), for 30 min at RT, which was chosen as the most suitable extraction procedure to proceed with the study (Table S1). Moreover, considering the initial moisture content of fresh *P. laurocerasus* leaves (58.02 ± 1.65%), no statistically significant difference was found between FLE and DLE in terms of prunasin content (Table S1).

#### 5.5.2. Determination of Total Cyanogenetic Glycosides by Colorimetric Assays

##### Picric Acid Method

The determination of the cyanogenetic glycosides by picric acid method is based on the enzymatic reaction by which β-glucosidase catalyzes the release of HCN, which reacts with picric acid on a paper test strip to produce 2,6-dinitro-5-hydroxy-4-hydroxylamino-1, 3 dicyclobenzene. This causes the paper strip to turn from yellow to brick red in proportion to the concentration of HCN produced. The test was carried out according to Appenteng et al. [14] with some modifications. 

First, to prepare the test paper, Whatman no. 2 filter papers were soaked with a 1.3% moistened picric acid in 2.5% *w*/*v* Na_2_CO_3_ solution and left to dry at RT. Strips of 3 cm × 0.6 cm are cut and fixed onto plastic strips measuring 5.5 cm × 0.7 cm, using vinyl glue. Different volumes (12.50, 25, 50 and 100 µL) of the reference standard prunasin (1 mg/mL in 70% ethanol) and laurel and cherry laurel extracts (25, 50 and 100 µL), prepared as reported in Section 5.5.1, were placed in vials with screw cap and brought to a volume of 100 µL with 70% ethanol. One-hundred microliters of 70% ethanol were used as blank. Fifty microliters of a β-glucosidase solution (5 U/mL) in phosphate buffer pH 6 was added to each vial, and the strips were placed inside, so that only the plastic edge was in contact with liquid. After this, they were capped and left to incubate at 37 °C for 16 h. The qualitative determination of cyanogenic glycosides (positive +/negative−) was carried out by comparing the color of the samples with the various concentrations of the reference standard prunasin (different shades of brick red) and with blank (yellow). The semi-quantitative determination was instead carried out by densitometric analysis using the open-source Image J v3.91 software (https://imagej.nih.gov/ij/download.html, accessed on 25 July 2022). Results, which represent the mean ± standard deviation of three independent experiments in triplicate (n = 3), were expressed as g of prunasin equivalents (PE)/100 g of fresh weight (FW).

##### Pyridine-Barbituric Acid Method

This spectrophotometric method is based on the ability of cyanides, once released in the form of HCN by the enzyme β-glucosidase, to react with chloramine T to form cyanogen chloride. This, in turn, reacts with pyridine to form N-cyanopyridinium chloride, which reacts with barbituric acid to form an intensely purple colored adduct, which can be determined spectrophotometrically at 585 nm. This test was carried out according to Kobaisy et al. [15] with some modifications. Different volumes (1.56, 3.13, 6.25, 12.50, and 25 µL) of the reference standard prunasin (1 mg/mL in 70% ethanol), laurel, and cherry laurel extracts (12.5 and 25 µL), prepared as reported in Section 5.5.1, and 100 µL of 70% ethanol as blank, were placed in 10 mL glass tubes with screw cap, brought to 100 µL with 70% ethanol and dried with nitrogen. After this, 500 µL of 0.1 M sodium acetate buffer (pH 6) were added and the solutions incubated with 50 µL of a β-glucosidase (5 U/mL) solution in buffer acetate (pH 5) for 1 h at 37 °C in a water bath. The reaction was stopped by adding 1 mL of 0.2 M NaOH. After 5 min at RT, the samples were neutralized with 0.95 mL of 0.2 M HCl up to pH 7. One milliliter of chloramine T (0.5%, *w*/*v*) was added to each test tube, incubating for 1 min at RT. Finally, 0.5 mL of an aqueous solution of pyridine-barbituric acid (barbituric acid: pyridine: HCl, 1:10:2, *w*/*v*/*v*, respectively) were added, leaving it to stand at RT for 4 min. The absorbance was recorded against the reaction blank at 585 nm by using an UV-Vis spectrophotometer (Shimadzu UV 1601, Kyoto, Japan). Results, which represent the mean ± standard deviation of three independent experiments in triplicate (n = 3), were expressed as g PE/100 g FW.

##### Determination of the Toxic Phytochemical Marker Prunasin by HPLC-DAD Analysis

The quali-quantitative determination of prunasin in the laurel and cherry laurel extracts, prepared as reported in the Section 5.5.1 and suitably diluted (5 and 1 mg/mL, respectively, in 70% ethanol), was carried out by a fast (15 min) high-pressure liquid chromatography analysis (HPLC Agilent 1100 series, Santa Clara, CA, USA) coupled with diode array detection (DAD, G1315). The isocratic elution was carried out, by a Luna Omega column 5 µm PS C18 100 Å 150 × 2.1 mm (Phenomenex, Torrance, CA, USA), using HPLC grade water (Solvent A, 90%) and acetonitrile (Solvent B, 10%) as mobile phase, both acidified with 0.1% HPLC-grade formic acid. The flow rate and column oven temperature were set at 0.4 mL/min and 40 °C, respectively. Five microliters of samples and reference standards, both filtered by 0.22 µm syringe filter, were injected. The UV-VIS spectra were recorded in the range 190–400 nm, whereas the acquisition was carried out at 220 nm, the maximum absorption wavelength of prunasin. The prunasin peak was identified by comparing the retention time and the UV-VIS spectrum of the analyte with that of the commercially available pure reference standard LC/MS-ELSD grade (purity ≥ 90%, Merck, Darmstadt, Germany). Quantification was performed using the external standard method by build an appropriate calibration curve (1.0–40 µg/mL). Results, which represent the mean ± standard deviation of three independent experiments in triplicate (n = 3), were expressed as g of prunasin/100 g FW. The analytical method was validated according to the to the current international guidelines [40] regarding selectivity, linearity, precision, robustness, limit of detection (LOD), limit of quantitation (LOQ), and recovery. LOD and LOQ values were calculated following the approach based on the standard deviation of the response and the slope of the calibration curves [40]. The validation parameters and relative results were reported in Table 1.

The method showed excellent linearity (R^2^ = 0.9999) in the wide range of tested concentrations (0.625–40 µg/mL), allowing to obtain an excellent sensitivity, as it is possible to observe from the values of the calculated detection and quantification limits (1.25 and 5 ng/mL for LOD and LOQ, respectively) (Table 1). Furthermore, the excellent recovery value (98.67%) and the low relative standard deviations (R.S.D.) found (Table 1), calculated both intra-day and inter-day, allow us to state that this is a precise, accurate, and reproducible method.

## Figures and Tables

**Table 1:** Validation of the HPLC-DAD analytical method.

Validation Parameters	Results
Calibration range (µg/mL)	0.625–40.0
Equation	y = 6.8292x
Linearity (R^2^)	0.9999
R.S.D. ^1^ (%), n = 6, within-day	0.158
R.S.D. (%), n = 6, between-day	0.187
LOD ^2^ (ng/mL)	1.25
LOQ ^3^ (ng/mL)	5.0
Recovery (%)	98.67

^1^ R.S.D., relative standard deviation; ^2^ LOD, limit of detection; ^3^ LOQ, limit of quantification.

## Data Availability

The data presented in this study are available in this article and supplementary material.

## Supplementary Materials

The following supporting information can be downloaded at: https://www.mdpi.com/article/10.3390/toxins14110726/s1, Table S1: Optimization of the extraction process carried out on fresh (FL) and dry (DL) leaves of *P. laurocerasus*. The numbers in bold represent the extraction conditions in which the highest prunasin content was obtained, as determined by HPLC-DAD analysis. The results represent the mean ± the standard deviation of three experiments in triplicate (n = 3).
