# Supplementary material for: Risk of Poisoning from Garden Plants: Misidentification between Laurel and Cherry Laurel
Source: Toxins (Basel). 2022 Oct 24;14(11):726. doi: 10.3390/toxins14110726 (PMC9697506; doi:10.3390/toxins14110726)
Supplement: Supplementary file 1 [file toxins-14-00726-s001.zip › toxins-1949002-supplementary.pdf]

Supplementary Materials: Risk of Poisoning from Garden Plants: Misidentification between Laurel and Cherry Laurel

Paola Malaspina, Federica Betuzzi, Mariarosaria Ingegneri, Antonella Smeriglio, Laura Cornara and Domenico Trombetta

Table S1. Optimization of the extraction process carried out on fresh (FL) and dry (DL) leaves of *P. laurocerasus*. The numbers in bold represent the extraction conditions in which the highest prunasin content was obtained, as determined by HPLC-DAD analysis. The results represent the mean ± the standard deviation of three experiments in triplicate (*n* = 3).

Temperature (°C)	Solvent (EtOH/H_2O, *v*/*v*)	Time (min)	Prunasin (g/100 g FLE*)	Prunasin (g/100 g DLE§)
25	90:10	15	2.56 ± 0.05	2.59 ± 0.03
25	90:10	30	2.84 ± 0.03	2.83 ± 0.02
25	90:10	60	2.98 ± 0.07	3.01 ± 0.03
25	80:20	15	3.12 ± 0.12	3.15 ± 0.05
25	80:20	30	3.21 ± 0.08	3.18 ± 0.10
25	80:20	60	3.43 ± 0.02	3.41 ± 0.04
25	70:30	15	3.65 ± 0.03	3.62 ± 0.05
**25**	**70:30**	**30**	**3.88 ± 0.04**	**3.85 ± 0.05**
25	70:30	60	3.54 ± 0.05	3.58 ± 0.01
25	60:40	15	3.33 ± 0.07	3.35 ± 0.05
25	60:40	30	3.28 ± 0.11	3.20 ± 0.06
25	60:40	60	3.10 ± 0.02	3.15 ± 0.08
25	50:50	15	2.76 ± 0.01	2.75 ± 0.04
25	50:50	30	2.82 ± 0.03	2.85 ± 0.06
25	50:50	60	2.88 ± 0.04	2.91 ± 0.05
30	90:10	15	2.82 ± 0.05	2.78 ± 0.06
30	90:10	30	2.94 ± 0.02	2.98 ± 0.04
30	90:10	60	3.04 ± 0.03	3.08 ± 0.04
30	80:20	15	3.21 ± 0.04	3.17 ± 0.05
30	80:20	30	3.28 ± 0.07	3.22 ± 0.08
30	80:20	60	3.32 ± 0.08	3.35 ± 0.05
30	70:30	15	3.55 ± 0.05	3.51 ± 0.06
30	70:30	30	3.62 ± 0.07	3.57 ± 0.05
30	70:30	60	3.60 ± 0.02	3.55 ± 0.08
30	60:40	15	2.45 ± 0.07	2.47 ± 0.04
30	60:40	30	2.54 ± 0.05	2.58 ± 0.06
30	60:40	60	2.51 ± 0.07	2.53 ± 0.09
30	50:50	15	2.21 ± 0.01	2.25 ± 0.06
30	50:50	30	2.30 ± 0.02	2.27 ± 0.04
30	50:50	60	2.35 ± 0.03	2.37 ± 0.02
60	90:10	15	2.65 ± 0.03	2.68 ± 0.05
60	90:10	30	2.71 ± 0.05	2.70 ± 0.06
60	90:10	60	2.80 ± 0.07	2.82 ± 0.04

60	80:20	15	2.83 ± 0.09	2.86 ± 0.05
60	80:20	30	2.95 ± 0.10	2.91 ± 0.04
60	80:20	60	3.04 ± 0.12	3.07 ± 0.07
60	70:30	15	3.21 ± 0.03	3.22 ± 0.02
60	70:30	30	3.34 ± 0.02	3.32 ± 0.03
60	70:30	60	3.15 ± 0.05	3.11 ± 0.06
60	60:40	15	2.54 ± 0.07	2.55 ± 0.04
60	60:40	30	2.65 ± 0.05	2.63 ± 0.06
60	60:40	60	2.72 ± 0.02	2.71 ± 0.01
60	50:50	15	2.23 ± 0.03	2.24 ± 0.02
60	50:50	30	2.30 ± 0.02	2.29 ± 0.02
60	50:50	60	2.38 ± 0.02	2.39 ± 0.01

[*]FLE, Fresh leaf extract; [§]DLE, dry leaf extract.
